# Efficacy and safety of Danlou tablets in traditional Chinese medicine for coronary heart disease: a systematic review and meta-analysis

**DOI:** 10.3389/fcvm.2023.1100006

**Published:** 2023-06-07

**Authors:** WeiLi Mao, Peng Lu, Renhong Wan, Kaili Mao, Yanzhu Lv, Jie Hu, Zhenling Fu, Jun Wang

**Affiliations:** ^1^ Department of Pharmacy, The Quzhou Affiliated Hospital of Wenzhou Medical University, Quzhou People’s Hospital, Quzhou, China; ^2^ Department of Pharmacy, Suzhou TCM Hospital Affiliated to Nanjing University of Chinese Medicine, Suzhou, China; ^3^ School of Acupuncture and Tuina, Tianjin University of Traditional Chinese Medicine, Tianjin, China

**Keywords:** Danlou tablet, coronary heart disease, randomized controlled trial, meta-analysis, complementary alternative therapy

## Abstract

**Background:**

Danlou tablets exert auxiliary advantages in treating coronary heart disease (CHD), but a summary of evidence-based proof is lacking. This study aims to systematically evaluate Danlou tablets in treating CHD from two aspects, including efficacy and safety.

**Methods:**

By a thorough retrieval of the four English databases, namely, PubMed, The Cochrane Library, Embase, and Web of Science, and the four Chinese databases, namely, CNKI, Wanfang, VIP database, and China Biomedical Literature Service System, we found all randomized controlled trials (RCTs) related to Danlou tablets in treating CHD. The retrieval time was from the construction of the database to April 2022. We engaged two researchers to screen the studies, extract the required data, and assess the risk of bias. We then used RevMan5.3 and STATA.14 software to conduct a meta-analysis. The Grading of Recommendations Assessment, Development, and Evaluation (GRADE) was used to evaluate the quality of outcome indicators.

**Results:**

Seventeen RCTs involving 1,588 patients were included. The meta-analysis results are displayed as follows: clinical treatment effect [risk ratio (RR) = 1.22, 95% confidence interval (CI): 1.16, 1.28, *P *< 0.00001], angina pectoris duration [MD = −0.2.15, 95% CI: −2.91, −1.04, *P *< 0.00001], angina pectoris frequency [standard mean difference (SMD) = −2.48, 95% CI: −3.42, −1.54, *P *< 0.00001], angina pectoris degree [SMD = −0.96, 95% CI: −1.39, −0.53, *P *< 0.0001], TC [MD = −0.71, 95% CI: −0.92, −0.51, *P *< 0.00001], TG [MD = −0.38, 95% CI: −0.53, −0.22, *P *< 0.00001], low-density lipoprotein cholesterol [MD = −0.64, 95% CI: −0.76, −0.51, *P *< 0.00001], high-density lipoprotein cholesterol [MD = 0.16, 95% CI: 0.11, 0.21, *P *< 0.00001], and adverse events [RR = 0.46, 95% CI: 0.24, 0.88, *P *= 0.02].

**Conclusion:**

The current evidence suggests that the combination of Danlou tablets and Western medicine can enhance the efficacy of CHD and does not increase adverse events. However, because of the limited number and quality of the included studies, the results of our study should be treated with caution. Further large-scale RCTs are necessary to verify the benefits of this approach.

## 1. Introduction

Cardiovascular and cerebrovascular diseases remain the dominant causes of death (1). Cardiovascular disease killed 17.9 million people worldwide in 2016, according to the World Health Organization, of which about 34% were due to coronary heart disease (CHD). An estimated 23.3 million cardiovascular deaths will occur in 2030 (2, 3). CHD seriously harms human health and aggravates the public health burden with its extremely high mortality (1). CHD is a heart disease caused by myocardial ischemia and hypoxia resulting from coronary atherosclerosis that causes lumen hardening, stenosis, or obstruction (4). CHD patients mainly present chest tightness, chest pain, angina pectoris, and other symptoms, which may cause sudden death in severe cases, significantly affecting patients' daily lives and even threatening their lives (5).

CHD is caused by a combination of personal constitution, genetic background, and environmental factors, and its symptoms involve multiple organs and tissues, eventually spreading to the whole body (6). In the development of the disease, different groups present different clinical characteristics due to differences in personal fitness, environment, genetic background, and other factors. Individualized therapy has become a future development trend in medicine (7). Traditional Chinese medicine (TCM) emphasizes individualized treatment, of which the TCM syndrome is the core of diagnosis and the key to treatment (6). According to the characteristics of various symptoms, CHD is mainly divided into blood stasis, phlegm turbidity, cold coagulation, qi deficiency, yin deficiency, and yang deficiency syndromes (8). For example, as a representative of Chinese patent medicine for blood stasis syndrome, Shexiang Baoxin pills can improve the curative effect of Western medicine in treating CHD according to evidence-based medical research (9) and are strongly recommended for treating CHD and angina pectoris in the prevention and treatment guidelines for CHD in China (10). Danlou tablets, a representative medicine of TCM in treating CHD with blood stasis and phlegm turbidity syndrome, have the effects of clearing qi, comforting the chest, relieving phlegm, dispersing the knot, invigorating blood circulation, and eliminating stasis (11). They can also significantly reduce atherosclerosis, ischemia, and reperfusion injury and improve myocardial dysfunction (12). Experimental studies have indicated that these tablets regulate oxidative stress, prevent inflammation, and reduce lipid deposition (13).

The results of a large-scale survey involving 5,284 patients with coronary disease displayed the top three TCM syndromes: blood stasis (79.3%), qi deficiency (56.5%), and phlegm turbidity (41.1%) (7). Compared with Shexiang Baoxin pills, Danlou tablets seem suitable for more CHD patients. However, in the *Clinical Application Guidelines for the Treatment of CHD of Chinese Patent Medicine (2020)*, the recommended intensity of Danlou tablets in treating CHD is weak (14). Therefore, more high-quality clinical studies and standardized systematic reviews on Danlou tablets in treating coronary heart disease are needed. Some clinical studies on Danlou tablets in treating coronary heart disease have been published. However, the design methods, efficacy evaluation criteria, and treatment courses of different studies are different. Therefore, our systematic evaluation aims to provide evidence-based support for the guidance of clinical and scientific medical work by evaluating the effectiveness and safety of Danlou tablets in treating CHD.

## 2. Methods

### 2.1. Study registration

This study was conducted per the guidance of the Preferred Reporting Items for Systematic Reviews and Meta-Analyses (PRISMA) (the checklist is shown in Supplementary Table S1). In addition, the protocol and registration information can be found at https://www.crd.york.ac.uk/prospero/#searchadvanced (registration number: CRD42021274916).

### 2.2. Inclusion criteria

(1) Research type: Randomized controlled trials (RCTs) were included.
(2) Diagnostic criteria: Patients diagnosed with CHD, which belongs to phlegm turbidity, blood stasis, or phlegm turbidity combined with blood stasis in TCM, were included. CHD diagnostic criteria refer to the *nomenclature and criteria for the diagnosis of ischemic heart disease* formulated by the WHO (15). The TCM syndrome diagnostic criteria refer to the *Guiding Principles for Clinical Research of TCM New Drugs*. There is no limit on nationality, race, age, gender, or disease duration.
(3) Interventions: The treatment group was treated orally with Danlou tablets combined with Western medicine, and the control group was given the same Western medicine alone or combined with Danlou tablets as a placebo.
(4) Outcome indicators: The outcomes are as follows:

(1) Primary outcomes: These involved clinical treatment effects. According to WHO criteria, the efficacy of CHD is divided into the following categories (5): Significantly effective: angina pectoris symptoms disappeared obviously and electrocardiogram (ECG) was normal; effective: angina pectoris symptoms improved to a certain extent and the ECG was improved; ineffective: angina symptoms were not relieved and the ECG was not changed. The clinical treatment effect represents the proportion of significantly and effectively effective patients to total patients.

(2) Secondary outcomes: These involved improvement of angina pectoris (including the frequency, duration, and pain degree of angina pectoris); it was defined as an improvement of angina pectoris only when the frequency, duration, and pain degree of angina pectoris were all improved. *Angina frequency* was defined as the number of angina attacks per day or week after treatment*.* The duration of angina pectoris was defined as the duration of each episode of angina pectoris after the end of treatment, measured in minutes. The degree of pain from angina should be evaluated as the angina score at the end of treatment. Blood lipid improvement [including total cholesterol (TC), triglyceride (TG), low-density lipoprotein cholesterol (LDL-C), and high-density lipoprotein cholesterol (HDL-C)] and the incidence of adverse events were also recorded.

### 2.3. Exclusion criteria

(1) Studies without diagnostic criteria or with diagnostic criteria errors;
(2) Studies without outcome indicators or with incorrect or incomplete data;
(3) Studies including patients after percutaneous coronary intervention (PCI); and
(4) Repetitive studies or studies with duplicate study data.

### 2.4. Search methods for identifying studies

The RCTs of Danlou tablets in treating CHD were comprehensively searched in Chinese and English databases from the database establishment to April 2021. Retrieval databases included PubMed, the Cochrane Library, CNKI, EMBASE, Web of Science, Wanfang, and VIP database. The search terms were “Danlou tablet,” “coronary diseases,” “coronary heart diseases,” “angina pectoris,” etc. Supplementary Table S2 shows the search strategy in PubMed.

### 2.5. Data collection and analysis

#### 2.5.1. Data extraction and management

Two researchers (PL and RW) screened the literature back to back, referring to the criteria above, and then cross-checked. A third researcher (WM) discussed the results after cross checking for any disagreement. After confirming the final included studies, we carefully read the complete study and extracted the required data.

The extracted data included the following: (1) general information (first author, publication year, samples in each group, baseline information, course of disease); (2) treatment protocol (name, medication frequency, and treatment course of oral drugs in each group); (3) risk bias assessment factors in RCTs; and (4) outcome indicators.

#### 2.5.2. Assessment of risk of bias

We assessed the risk bias according to the RCT risk assessment tool recommended by the Cochrane Collaboration Handbook. Two researchers (PL and RW) completed risk bias assessments for each study. After completion, they cross-checked the data, and discussion was required in case of differences. If no agreement could be reached, negotiation with a third researcher (WM) was performed, and a final consensus would be reached.

#### 2.5.3. Data synthesis

RevMan5.3 software was used for data synthesis. The dichotomous variables were expressed as a relative risk ratio (RR); for continuous outcomes, the weighted mean difference (WMD) and standard mean difference (SMD) were used as effect sizes for consistency and inconsistency between measuring tools and measuring units, respectively. All were presented as 95% confidence intervals (CIs). We used *χ*^2^ and *I*^2^ values to determine the heterogeneity. *P* ≥ 0.1, *I*^2^ ≤ 50% indicated low heterogeneity, and we chose a fixed-effects model. *P *< 0.1, *I*^2^*^ ^*> 50% indicated significant heterogeneity, and then the heterogeneity was analyzed. We used subgroup or sensitivity analysis to explore the origin of heterogeneity and then used a random-effects model for merging after excluding apparent clinical and methodological heterogeneity. As recommended in the Cochrane manual, one-by-one elimination would be used in the sensitivity analysis to test the stability of meta-analysis results of indicators. For the primary outcome indicators, the publication bias was qualitatively detected by a funnel plot, and the potential publication bias was quantitatively evaluated by Egger's and Begg's tests.

#### 2.5.4. Evidence quality evaluation

Two researchers (PL and RW) graded the outcome indicators by the Grading of Recommendation, Development, and Evaluation (GRADE) (16). Similarly, after completing the assessment, the two researchers cross-checked with each other, discussed with the third researcher (WM) for any disagreement, and finally reached a consensus.

## 3. Results

### 3.1. Studies' characteristics

**Figure 1:**
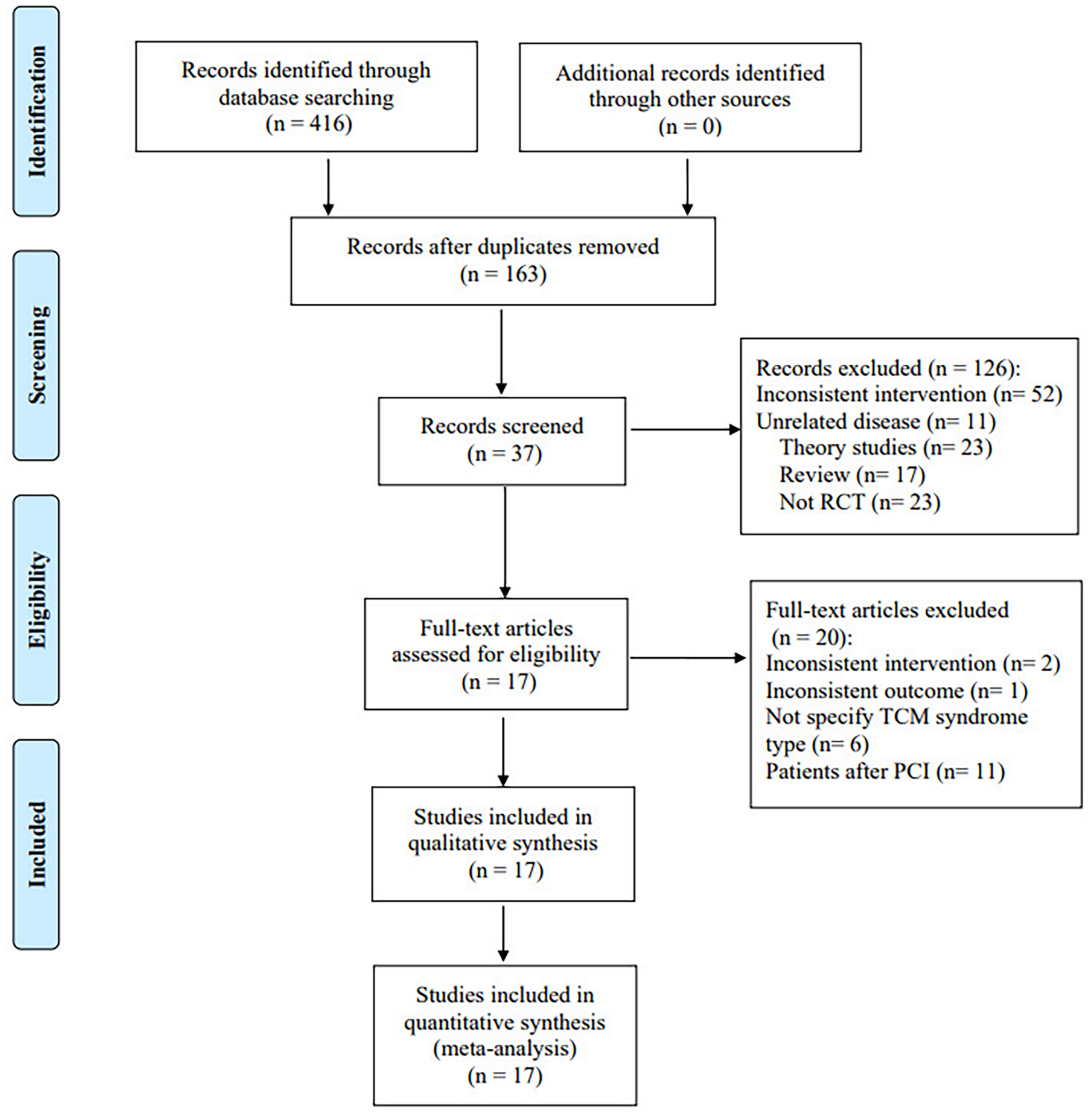
Flow diagram.

A total of 416 pieces of related literature were obtained after preliminary examination, and 163 were obtained after eliminating duplication, of which 126 were excluded after reading titles and abstracts, and 37 remained. The full text of 37 studies was read, and 20 articles were excluded. Finally, 17 studies were included. Among the 20 excluded articles, 11 included patients after PCI as intervention objects, 6 did not specify the TCM syndrome type, 2 did not conform to the intervention plan, and 1 did not have the required outcome indicators. Figure 1 depicts a flow diagram of the literature screening process.

The essential characteristics of the included studies are presented in Table 1. Patients in the treatment group took Danlou tablets combined with Western medicine, and patients in the control group took only Western medicine. The characteristics of the intervention measures are presented in Table 2.

**Table 1:** Basic characteristics of the included studies.

Study cohort	No (T/C)	Gender		Age		Course (day)	Outcome
		T (M/W)	C (M/W)	T	C		
Su B 2021 (17)	100/100	51/49	52/48	61.66 ± 5.52	61.59 ± 5.67	28	(1)
Tan JY 2013 (18)	60/60	39/21	39/21	62.5 ± 7	60.1 ± 9	28	(1)(2)(3)(4)
Tang RK 2013 (19)	20/20	13/7	11/9	62 ± 9.38	61.05 ± 8.55	180	(1)(2)(3)(4)(5)
Wang L 2015 (20)	30/30	18/12	16/14	64.25 ± 3.35	66.99 ± 4.11	28	(1)(2)(3)(4)(5)
Wang SH 2012 (21)	32/30	25/7	24/6	60.2 ± 9.0	62.7 ± 7.1	28	(1)(2)(3)(4)
Wang WL 2021 (22)	43/43	22/21	20/23	69.93 ± 2.04	69.84 ± 1.96	28	(2)(3)
Wang YH 2018 (23)	35/35	20/15	18/17	55.8 ± 8. 9	56.5 ± 9.2	28	(2)(3)
Wei Q 2015 (24)	40/30	22/23	24/21	52.77 ± 9.67	51.82 ± 8.86	28	(1)(2)(3)(4)(5)(6)(7)(8)(9)
Xing XH 2020 (25)	60/60	33/27	34/32	56.53 ± 5.44	55.91 ± 5.13	56	(1)(5)
Zang GP 2018 (26)	53/53	40/13	38/15	48.88 ± 5.01	49.94 ± 4.69	30	(1)(2)(3)
Ma XF 2017 (27)	40/40	28,12	25/15	58.5 ± 8.5	59.5 ± 8.5	28	(5)
Ren DZ 2014 (28)	34/34	20/14	18/16	61.2	63.5	30	(1)(5)(6)(7)(8)(9)
Tian CH 2017 (29)	47/47	28/21	27/20	58.8 ± 3.5	59.1 ± 3.5	30	(1)
Wang M 2019 (30)	50/50	28/22	29/21	60.32 ± 7.65	60.81 ± 7.94	28	(1)(5)
Yang XY 2014 (31)	41/41	23/18	28/13	—	—	30	(1)(2)(3)(4)
Zang JH 2018 (32)	45/45	22/23	24/21	60.23 ± 8.12	60.06 ± 7.58	180	(1)(6)(7)(8)(9)
Zhou MJ 2013 (33)	70/70	—	—	—	—	30	(1)(2)(3)(4)

Outcome: (1) clinical treatment effect; (2) angina pectoris frequency; (3) angina pectoris duration; (4) angina pectoris degree; (5) adverse events; (6) TG; (7) LDL-C; (8) HDL-C; and (9) TC.

**Table 2:** Characteristics of the intervention measures.

Study	Interventions of the treatment group		Interventions of the control group
	Chinese medicine	Western medicine	Western medicine
Su B 2021 (17)	Danlou tablet 1.2 g, tid	Aspirin 300 mg/time, tid;	Aspirin 300 mg/time, tid;
		Atorvastatin, 20 mg/time, qd	Atorvastatin, 20 mg/time, qd
Tan JY 2013 (18)	Danlou tablet 1.5 g, bid	Aspirin 100 mg, qd;	Aspirin 100 mg, qd;
		Simvastatin 20 mg, qd;	Simvastatin 20 mg, qd;
		Isosorbide nitrate 10 mg, tid	Isosorbide nitrate 10 mg, tid
Kang RK 2013 (19)	Danlou tablet 1.5 g, tid	Hypoglycemic,	Hypoglycemic,
		Lipid-regulating	Lipid-regulating
Wang L 2015 (20)	Danlou tablet 1.5 g, tid	Bayaspirin 100 mg, qd;	Bayaspirin 100 mg, qd;
		Atorvastatin calcium 20 mg, qd	Atorvastatin calcium 20 mg, qd
		Polyvir, 75 mg, qd;	Polyvir, 75 mg, qd;
		Low-molecular-weight heparin calcium 6000 iu, bid, subcutaneous injection;	Low-molecular-weight heparin calcium 6000 iu, bid, subcutaneous injection;
		Metoprolol succinate 23.75–47.5 mg, qd	Metoprolol succinate 23.75–47.5 mg, qd
Wang SH 2012 (21)	Danlou tablet 1. 5 g, bid	Aspirin 100 mg, qd;	Aspirin 100 mg, qd;
		Simvastatin 20 mg, qd;	Simvastatin 20 mg, qd;
		Isosorbide nitrate 10 mg, tid	Isosorbide nitrate 10 mg, tid
Wang WL 2021 (22)	Danlou tablet 1.5 g, bid	Aspirin sustained-release tablets;	Aspirin sustained-release tablets;
		Isosorbide mononitrate 20 mg, bid	Isosorbide mononitrate 20 mg, bid
Wang YH 2018 (23)	Danlou tablet 1.5 g, bid	Aspirin 100 mg, qd;	Aspirin 100 mg, qd;
		`Rosuvastatin 10 mg, qd;	Rosuvastatin 10 mg, qd;
		Isosorbide nitrate 10 mg, tid	Isosorbide nitrate 10 mg, tid
Wei Q 2015 (24)	Danlou tablet 1.5 g, bid	Aspirin enteric-coated tablet/Bayaspirin 100 mg, qd;	Aspirin enteric-coated tablet/Bayaspirin 100 mg, qd;
		Clopidogrel 75 mg, qd;	Clopidogrel 75 mg, qd;
		Simvastatin 20 mg, qd;	Simvastatin 20 mg, qd;
		Isosorbide nitrate 10 mg, tid	Isosorbide nitrate 10 mg, tid
		Metoprolol tartrate tablets 25 mg, bid	Metoprolol tartrate tablets 25 mg, bid
Xing XH 2020 (25)	Danlou tablet 1.5 g, tid	Statins,	Statins,
		coronary vasodilators	coronary vasodilators
Zang GP 2018 (26)	Danlou tablet 1.5 g, tid	Aspirin tablet 500 mg, qd;	Aspirin tablet 500 mg, qd;
		Atorvastatin calcium 20 mg, qd;	Atorvastatin calcium 20 mg, qd;
		Isosorbide mononitrate sustained-release tablets 60 mg, qd	Isosorbide mononitrate sustained-release tablets 60 mg, qd
Ma XF 2017 (27)	Danlou tablet 1.5 g, tid	Isosorbide mononitrate tablets 20 mg, tid	Isosorbide mononitrate tablets 20 mg, tid
Ren DZ 2014 (28)	Danlou tablet 1.2 g, tid	Aspirin; low-molecular-weight heparin calcium; nitrates; *β*blockers	Aspirin; low-molecular-weight heparin calcium; nitrates; *β*-blockers
Tian CH 2017 (29)	Danlou tablet 2.5 g, tid	Trimetazidine hydrochloride tablets 20 mg, tid	Trimetazidine hydrochloride tablets 20 mg, tid
Wang M 2019 (30)	Danlou tablet 1.5 g, qd	Aspirin 100 mg, qd;	Aspirin 100 mg, qd;
		Atorvastatin calcium 20 mg, qd	Atorvastatin calcium 20 mg, qd
Yang XY 2014 (31)	Danlou tablet 0. 5–1 g, bid	Aspirin 150 mg, qd;	Aspirin 150 mg, qd;
		Atorvastatin 10 mg, qd	Atorvastatin 10 mg, qd
Zang JH 2018 (32)	Danlou tablet 1.5 g, tid	Simvastatin pills 10 mg, qd	Simvastatin pills 10 mg, qd
Zhou MJ 2013 (33)	Danlou tablet 0. 5–1 g, qd or bid	Aspirin 150 mg, qd;	Aspirin 150 mg, qd;
		Atorvastatin 10 mg, qd	Atorvastatin 10 mg, qd

### 3.2. Risk of bias assessment

Among the 17 RCTs, 10 (17–26) were randomly assigned by the random number table method and rated as low risk, while the rest (27–33) did not describe the method of random sequence generation and were rated as unclear; only one (20) used double-blind and placebo controls, and the use of blindness and allocation hiding was rated as low risk, while the rest (17–19, 21–33) did not mention the use of blindness and allocation hiding and were rated as unclear; none of the studies (17–33) reported outcome indicators with incomplete information, and none of them had a selective reporting; thus, their risk rating was evaluated as low. For other biases, all (17–33) were evaluated as unclear. The bias risk assessment results are presented in Table 3.

**Table 3:** Risk of bias summary.

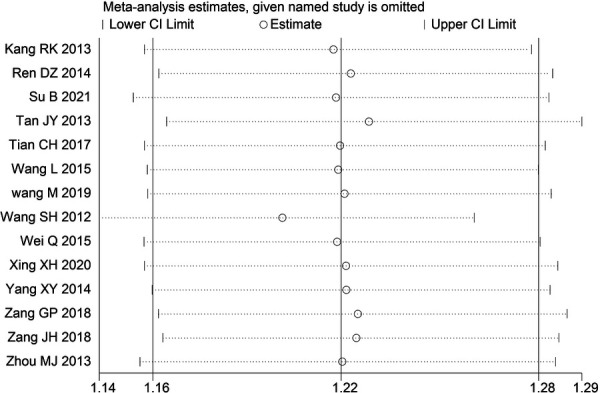

### 3.3. Outcome indicators

#### 3.3.1. Clinical treatment effect

Fourteen RCTs (17–21, 24–26, 28–33), including 1,352 patients, reported the effectiveness of clinical treatment, and heterogeneity test results indicated no heterogeneity (*P *= 0.95, *I*^2^*^ ^*= 0%). By using a fixed-effects model, meta-analysis results showed a better effect in the treatment group than in the control group [(RR = 1.22, 95% CI: 1.16, 1.28, *P *< 0.00001)] (*P *< 0.05, Table 4). Due to the difference in the daily dose of Danlou tablets among the patients, we divided them into three subgroups, <3, ≥3, and <4.5, ≥4.5 g, according to the daily dose. Heterogeneity test results indicated no significant heterogeneity of the three subgroups: <3 g (*P *= 0.99, *I*^2^*^ ^*= 0%), ≥3 and <4.5 g (*P *= 0.25, *I*^2^*^ ^*= 26%), and ≥4.5 g (*P *= 0.99, *I*^2^*^ ^*= 0%). By using a fixed-effects model, the results are as follows: <3 g [(RR = 1.21, 95% CI: 1.10, 1.32, *P *< 0.0001)], ≥3 and <4.5 g [(RR = 1.24, 95% CI: 1.14, 1.35, *P *< 0.00001)], and ≥4.5 g [(RR = 1.20, 95% CI: 1.11, 1.30, *P *< 0.0001)], indicating a better effect in the treatment group than in the control group in three subgroups (Table 5).

**Table 4:** Meta-analysis of the effectiveness of clinical treatment.

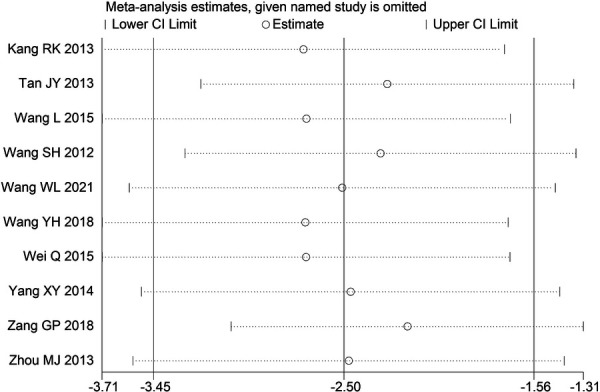

**Table 5:** Subgroup analysis of clinical treatment effects.

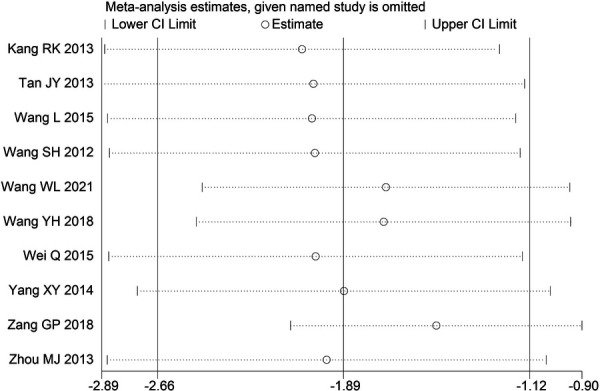

#### 3.3.2. Duration of angina pectoris

Ten RCTs (18–24, 26, 31, 33), including 836 patients, reported the angina pectoris duration, and the results of the heterogeneity test suggested significant heterogeneity (*P *< 0.00001, *I*^2^*^ ^*= 96%). We explored the source of heterogeneity through sensitivity analysis. The exclusion of any study had no significant effect on the heterogeneity results, indicating that interstudy heterogeneity did not affect the results, so we used a random-effects model to combine them. Results displayed lower angina pectoris duration in the treatment group than that in the control group [(MD = −0.2.15, 95% CI: −2.91, −1.04, *P *< 0.00001), Table 6]. In terms of the treatment course, among all the included studies, the treatment course in Tang (19) was 180 days, while it was 28 or 30 days in other studies. After excluding Tang (19), *I*^2^ was found to be 96%, and the heterogeneity did not change significantly. We divided them into three subgroups, <3, ≥3 and <4.5, and ≥4.5 g (Table 7), according to the daily dose of the Danshen tablet. Results showed significant intergroup heterogeneity of the three subgroups: <3 g (*P *= 0.03, *I*^2^*^ ^*= 79%), ≥3 and <4.5 g (*P *< 0.00001, *I*^2^*^ ^*= 87%), and ≥4.5 g (*P *< 0.00001, *I*^2^*^ ^*= 87%). Results from a random-effect model are as follows: <3 g [(MD = −3.60, 95% CI: −4.90, −2.30, *P *< 0.0001)], ≥3 and <4.5 g [(MD = −1.86, 95% CI: −2.45, −1.27, *P *< 0.00001)], and ≥4.5 g [(MD = −1.72, 95% CI: −4.17, 0.73, *P *= 0.17)], indicating lower angina pectoris duration in the treatment group than that in the control group when the daily dose of Danlou tablets was <4.5 g (*P *< 0.05). There was no difference when the daily dose of Danlou tablets was more significant than or equal to 4.5 g (*P* > 0.05).

**Table 6:** Meta-analysis of the duration of angina pectoris.

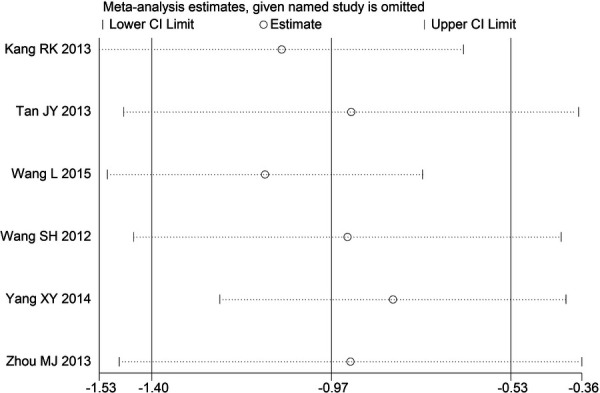

**Table 7:** Subgroup analysis of the duration of angina pectoris.

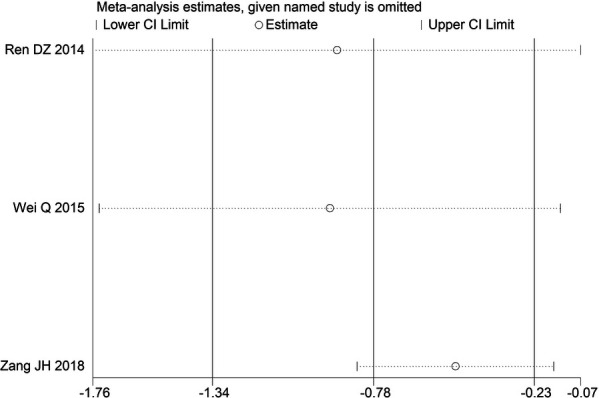

#### 3.3.3. Frequency of angina pectoris

Ten RCTs (18–24, 26, 31, 33), including 836 patients, reported the frequency of angina pectoris, and the results of the heterogeneity test suggested significant heterogeneity (*P *< 0.00001, *I*^2^*^ ^*= 96%). We explored the source of heterogeneity through sensitivity analysis. The exclusion of any study had no prominent effect on the heterogeneity results, indicating no effect of interstudy heterogeneity on the results, so we combined them through a random-effects model. Since the measurement units of angina pectoris frequency differed in different research centers, SMD was used as a valid indicator for meta-analysis. Results indicated lower angina pectoris frequency in the treatment group than in the control group [(SMD = −2.48, 95% CI: −3.42, −1.54, *P *< 0.00001), Table 8]. In terms of the treatment course, among all the included studies, the treatment course in Tang (19) was 180 days, while in other studies, it was 28 or 30 days. After excluding Tang (19), *I*^2^ was found to be 96%, and the heterogeneity did not change significantly. We divided them into three subgroups: <3, ≥3 and <4.5, and ≥4.5 g subgroups (Table 9) according to the daily dose of the Danshen tablet. Heterogeneity test results showed a loss of heterogeneity in the <3 g subgroup (*P *= 0.87, *I*^2^*^ ^*= 0%) and significant heterogeneity in the ≥3 and <4.5 g subgroup (*P *< 0.00001, *I*^2^*^ ^*= 97%) and in the ≥4.5 g subgroup (*P *< 0.00001, *I*^2^*^ ^*= 96%). Meta-analysis results, through a random-effects model, revealed that: <3 g [(SMD = −2.75, 95% CI: −3.12, −2.38, *P *< 0.0001)], ≥3 and <4.5 g [(SMD = −2.50, 95% CI: −3.94, −1.07, *P *= 0.0006)], and ≥4.5 g [(SMD = −2.27, 95% CI: −4.88, 0.33, *P *= 0.09)], indicating lower angina pectoris frequency in the treatment group than the control group when the daily dose of Danlou tablets was <4.5 g (*P *< 0.05), and there was no difference when the daily dose of Danlou tablets was ≥4.5 g (*P* > 0.05).

**Table 8:** Meta-analysis of the frequency of angina pectoris.

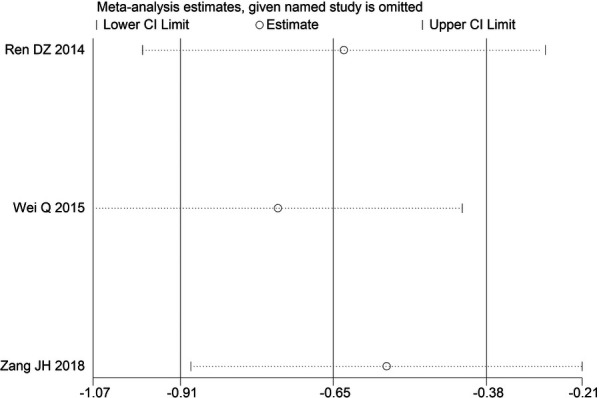

**Table 9:** Subgroup analysis of angina pectoris frequency.

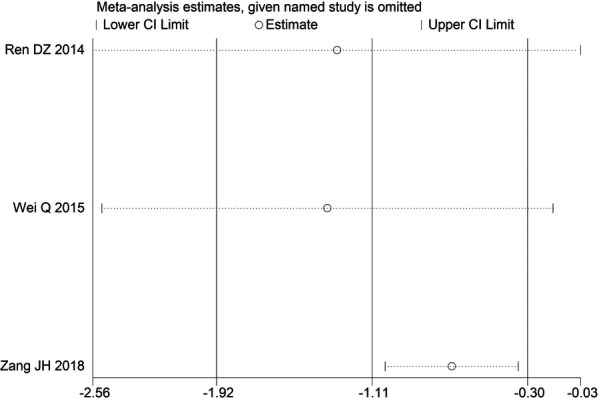

#### 3.3.4. Degree of angina pectoris

Six RCTs (18–21, 25, 33), including 504 patients, reported the angina pectoris degree (*P *= 0.0001, *I*^2^*^ ^*= 80%). We explored the source of heterogeneity through sensitivity analysis. The exclusion of any study had no significant effect on the heterogeneity results, indicating that interstudy heterogeneity did not affect the result; thus, we combined them through a random-effect model. Since the measurement units of the degree of angina pectoris differed in different research centers, SMD could be used as a valid indicator in meta-analysis. Results displayed a lower angina pectoris degree in the treatment group vs. the control group [(SMD = −0.96, 95% CI: −1.39, −0.53, *P *< 0.0001), Table 10].

**Table 10:** Meta-analysis of the degree of angina pectoris.

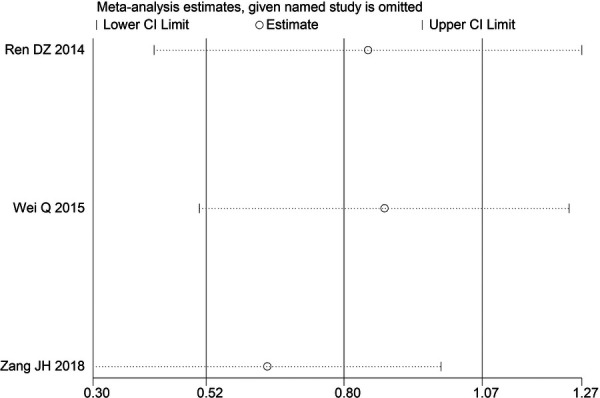

#### 3.3.5. Total cholesterol

Three RCTs (24, 28, 32) including 228 patients reported TC. No heterogeneity could be seen from the heterogeneity test results (*P *= 0.63, *I*^2^*^ ^*= 0%). Through a fixed-effect model, the results displayed a lower TC for the treatment group than that for the control group [(MD = −0.71, 95% CI: −0.92, −0.51, *P *< 0.00001), Table 11].

**Table 11:** Meta-analysis of TC.

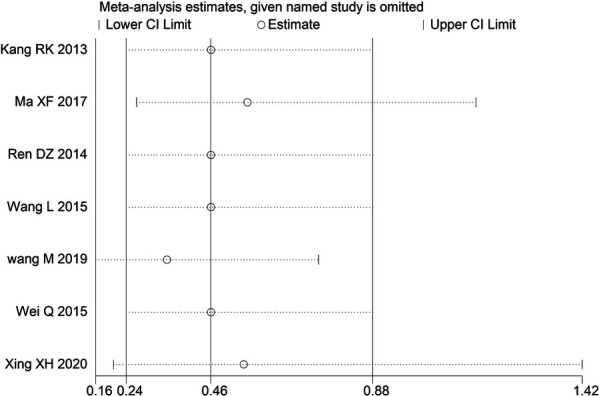

#### 3.3.6. Triglyceride

Three RCTs (24, 28, 32), including 228 patients, reported TG. No heterogeneity could be seen from the heterogeneity test results (*P *= 0.47, *I*^2^*^ ^*= 0%). Through a fixed-effect model, the results displayed a lower TG for the treatment group than that for the control group [(MD = −0.38, 95% CI: −0.53, −0.22, *P *< 0.00001), Table 12].

**Table 12:** Meta-analysis of TG.

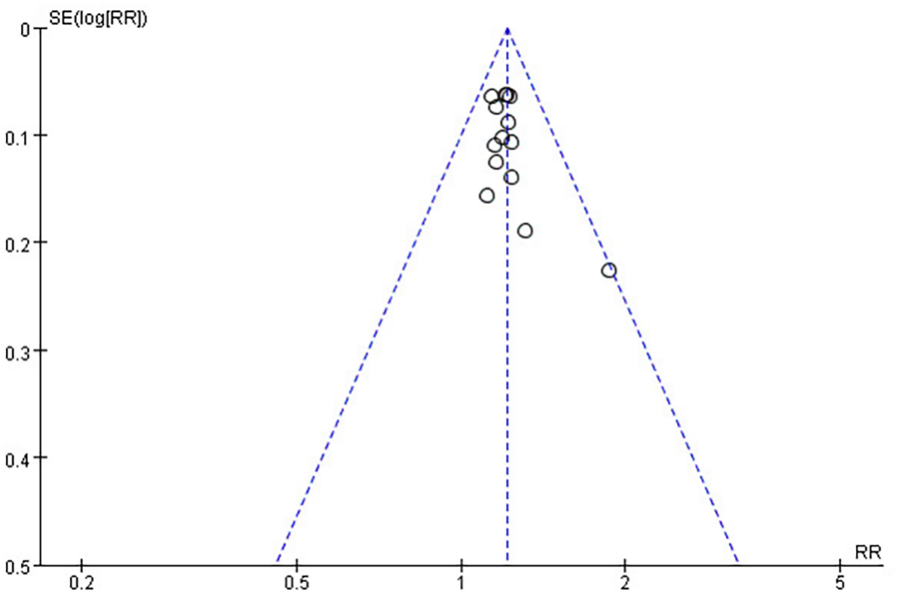

#### 3.3.7. Low-density lipoprotein cholesterol

Three RCTs (24, 28, 32), including 228 patients, reported LDL-C. No heterogeneity could be seen from the heterogeneity test results (*P *= 0.50, *I*^2^*^ ^*= 0%). Through a fixed-effect model, the results displayed lower LDL-C for the treatment group than that for the control group [(MD = −0.64, 95% CI: −0.76, −0.51, *P *< 0.00001), Table 13].

**Table 13:** Meta-analysis of LDL-C.

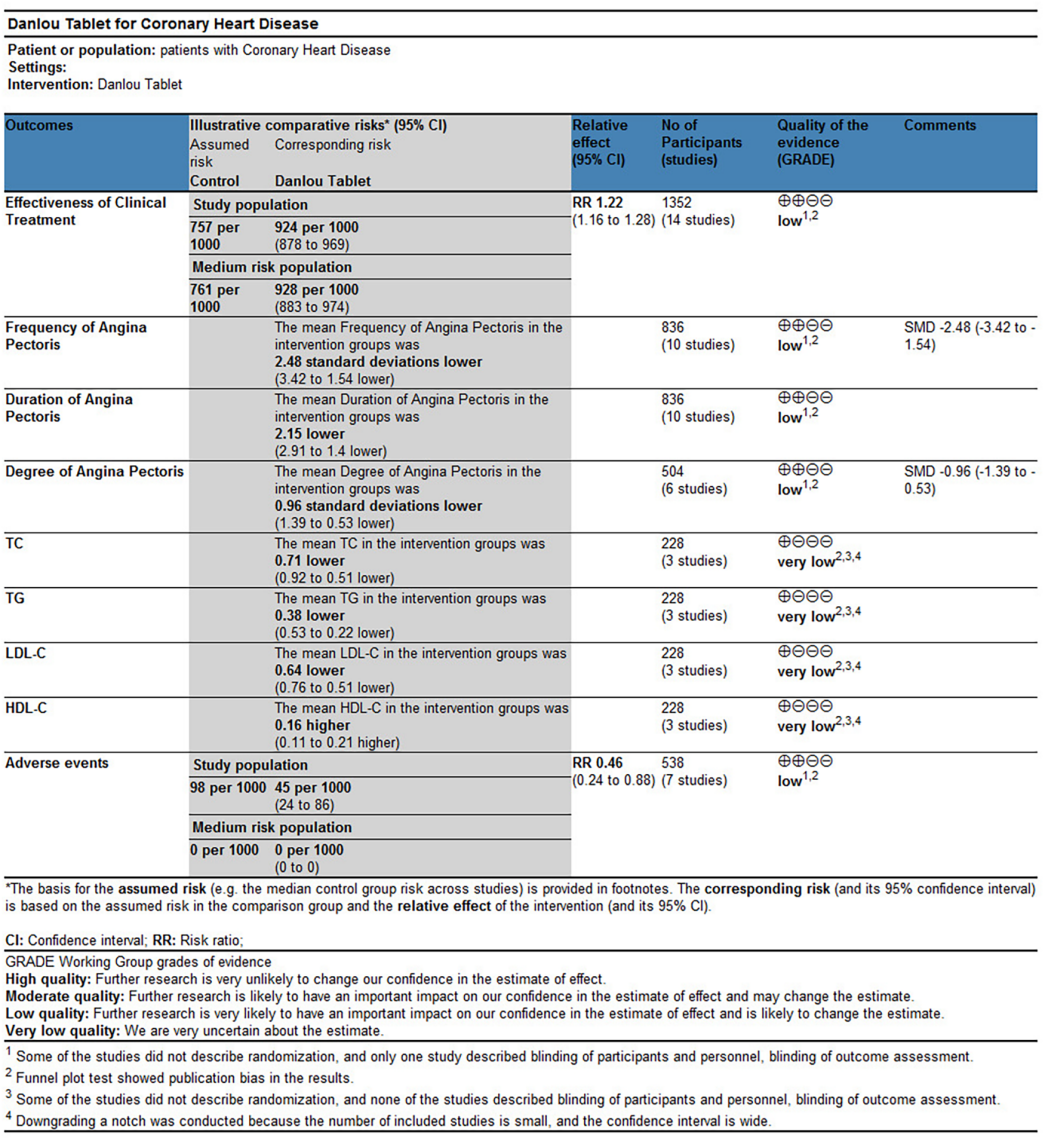

#### 3.3.8. High-density lipoprotein cholesterol

Three RCTs (24, 28, 32), including 228 patients, reported HDL-C. No heterogeneity could be seen from the heterogeneity test results (*P *= 0.44, *I*^2^*^ ^*= 0%). Through a fixed-effect model, results displayed higher HDL-C for the treatment group than that for the control group [(MD = 0.16, 95% CI: 0.11, 0.21, *P *< 0.00001), Table 14].

**Table 14:** Meta-analysis of HDL-C.

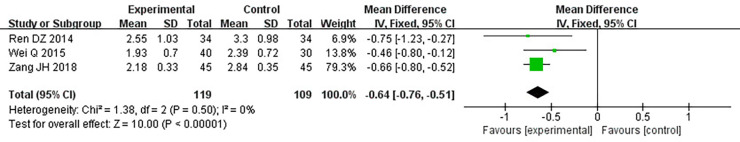

#### 3.3.9. Adverse events

Seven RCTs (19, 20, 24, 25, 27, 28, 30), including 538 patients, reported an adverse event rate, of which four RCTs (19, 20, 24, 28) showed no adverse events in any of the groups. Meta-analysis results of adverse events reported in the remaining three RCTs (25, 27, 30) are presented in Table 15, indicating fewer adverse events in the treatment group than those in the control group [(RR = 0.46, 95% CI: 0.24, 0.88, *P *= 0.02), Table 15]. The proportion of adverse events is shown in Table 16.

**Table 15:** Meta-analysis of adverse events.

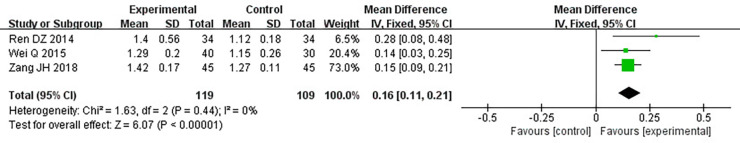

**Table 16:** Proportion of the adverse events.

Adverse event	Treatment group (*n* = 274)	Control group (*n* = 264)
Abnormal blood routine [*n* (%)]	1 (0.0036)	4 (0.0152)
Abdominal distension [*n* (%)]	1 (0.0036)	4 (0.0152)
Diarrhea [*n* (%)]	4 (0.0146)	5 (0.0189)
Nausea, vomiting [*n* (%)]	2 (0.0073)	4 (0.0152)
Thirst [*n* (%)]	0 (0)	2 (0.0076)
Abnormal liver function [*n* (%)]	1 (0.0036)	3 (0.0114)
Erythra [*n* (%)]	2 (0.0073)	2 (0.0076)
Insomnia [*n* (%)]	1 (0.0036)	1 (0.0038)
Add up [*n* (%)]	8 (0.0292)	23 (0.0871)

### 3.4. Subgroup analysis

We conducted a subgroup analysis based on the course of treatment (Table 17). In the case of the clinical treatment effect, we used a fixed-effect model, which showed greater efficacy than the control group in the subgroups of 28, 30, and 56 days. However, the 180-day subgroup showed no difference in efficacy between the treatment and control groups [28 days: (RR = 1.24, 95% CI: 1.16, 1.34, *P < *0.00001); 30 days: (RR = 1.19, 95% CI: 1.11, 1.29, *P < *0.00001); 56 days: (RR = 1.20, 95% CI: 1.06, 1.36, *P *= 0.0003); 180 days: (RR = 1.20, 95% CI: 0.99, 1.44, *P* = 0.06)]. In the case of the duration of angina pectoris, we used a random-effects model, which showed greater efficacy than that of the control group in the subgroups of 28 and 30 days, but the 180-day subgroup showed no difference in efficacy between the treatment and control groups [28 days: (MD = −1.69, 95% CI: −2.29, −1.09, *P* < 0.00001); 30 days: (MD = −3.69, 95% CI: −4.33, −3.05, *P* < 0.00001); 180 days: (MD = −0.40, 95% CI: −1.15, 0.35, *P* = 0.30)]. In the case of the frequency of angina pectoris, we used a random-effects model, and the results indicated that each subgroup showed greater efficacy than the control group [28 days: (SMD = −2.22, 95% CI: −3.43, −1.01, *P* = 0.0003); 30 days: (SMD = −3.59, 95% CI: −5.02, −2.16, *P* < 0.0001); 180 days: (SMD = −0.67, 95% CI: −1.31, −0.03, *P* = 0.04%)]. In the case of the degree of angina pectoris, we used a random-effect model. The results showed that the subgroups with 28 and 30 days of treatment showed greater efficacy than the control group. However, the 180-day subgroup showed no difference in efficacy between the treatment and control groups [28 days: (SMD = −0.84, 95% CI: −1.45, −0.22, *P* = 0.008); 30 days: (SMD = −1.42, 95% CI: −2.01, −0.83, *P* < 0.00001); 180 days: (SMD = −0.26, 95% CI: −0.89, 0.36, *P* = 0.41)].

**Table 17:** Subgroup analysis results.

Outcome index	Course (days)	Number of studies	Heterogeneity		Meta-analysis results		
			*I* ^2^	*P*	Model	95%CI	*P*
Clinical treatment effect	28	6	7%	0.37	RR Fixed effect	**1.24** (**1.16, 1.34)**	**<0**.**00001**
	30	5	0%	0.99		**1.19** (**1.11, 1.29)**	**<0**.**00001**
	56	1	NA	NA		**1.20** (**1.06, 1.36)**	**0**.**003**
	180	2	0%	0.56		1.20 (0.99, 1.44)	0.06
Duration of angina pectoris	28	6	89%	<0.00001	MD Random effect	**−1.69**(**−2.29, −1.09)**	**<0**.**00001**
	30	3	67%	0.05		**−3.69**(**−4.33, −3.05)**	**<0**.**00001**
	180	1	NA	NA		−0.40(−1.15, 0.35)	0.30
Frequency of angina pectoris	28	6	96%	<0.00001	SMD Random effect	**−2.22**(**−3.43, −1.01)**	**0**.**0003**
	30	3	94%	<0.00001		**−3.59**(**−5.02, −2.16)**	**<0**.**00001**
	180	1	NA	NA		**−0.67**(**−1.31, −0.03)**	**0**.**04**
Degree of angina pectoris	28	3	80%	0.006	SMD Random effect	**−0.84**(**−1.45, −0.22)**	**0**.**008**
	30	2	72%	0.06		**−1.42**(**−2.01, −0.83)**	**<0**.**00001**
	180	1	NA	NA		−0.26(−0.89, 0.36)	0.41

NA, data unavailable; MD, mean difference; SMD, standard mean difference; CI, confidence interval. The bold font indicates a statistically significant difference between the two treatments.

### 3.5. Sensitivity analysis

**Figure 2:**
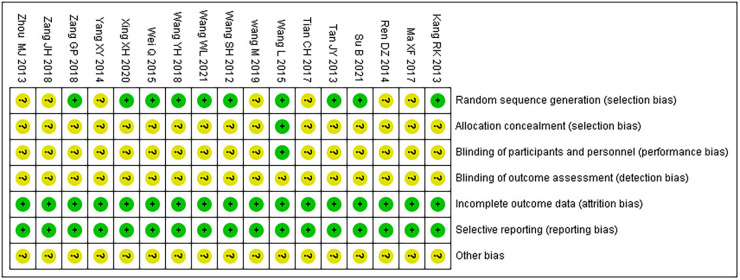
Sensitivity analysis of the effectiveness of clinical treatment.

**Figure 3:**
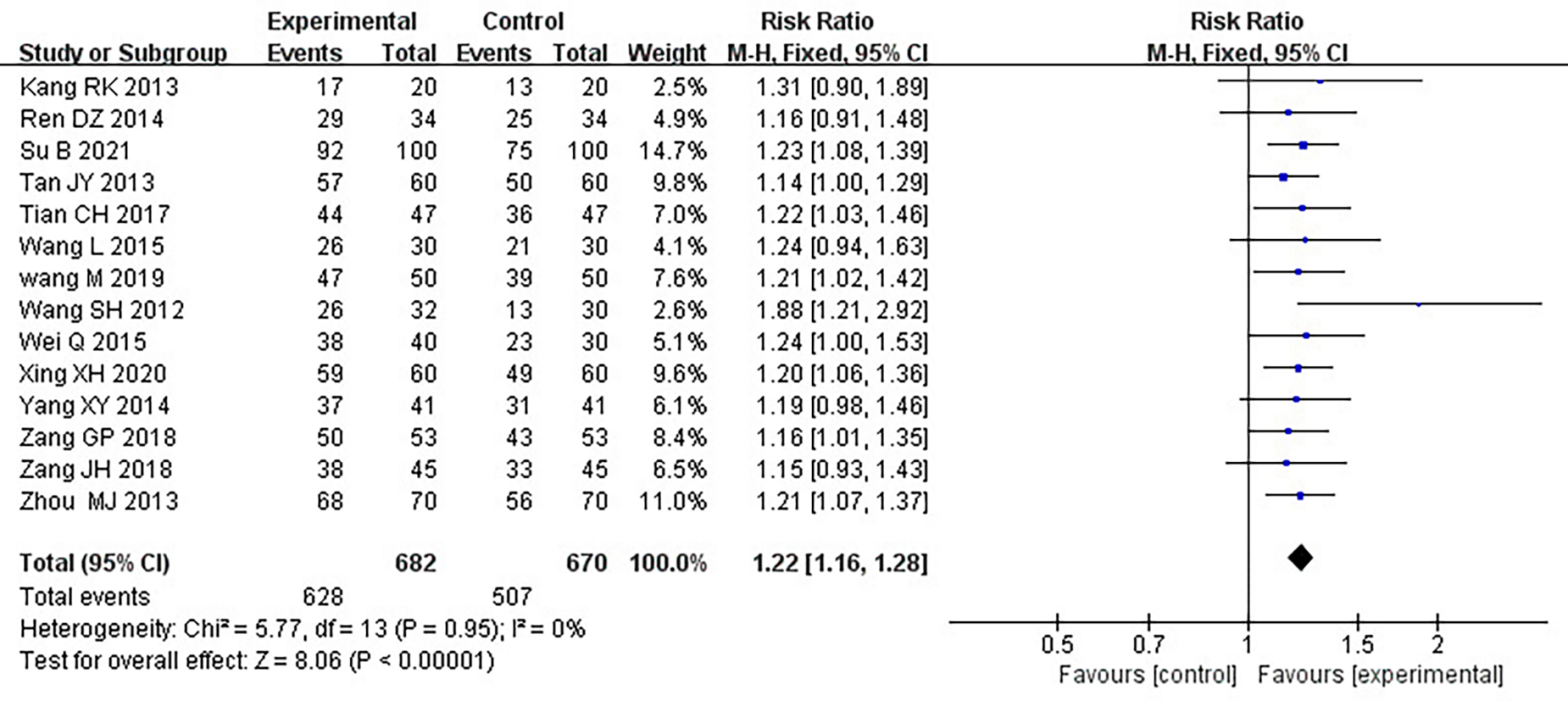
Sensitivity analysis of the frequency of angina pectoris.

**Figure 4:**
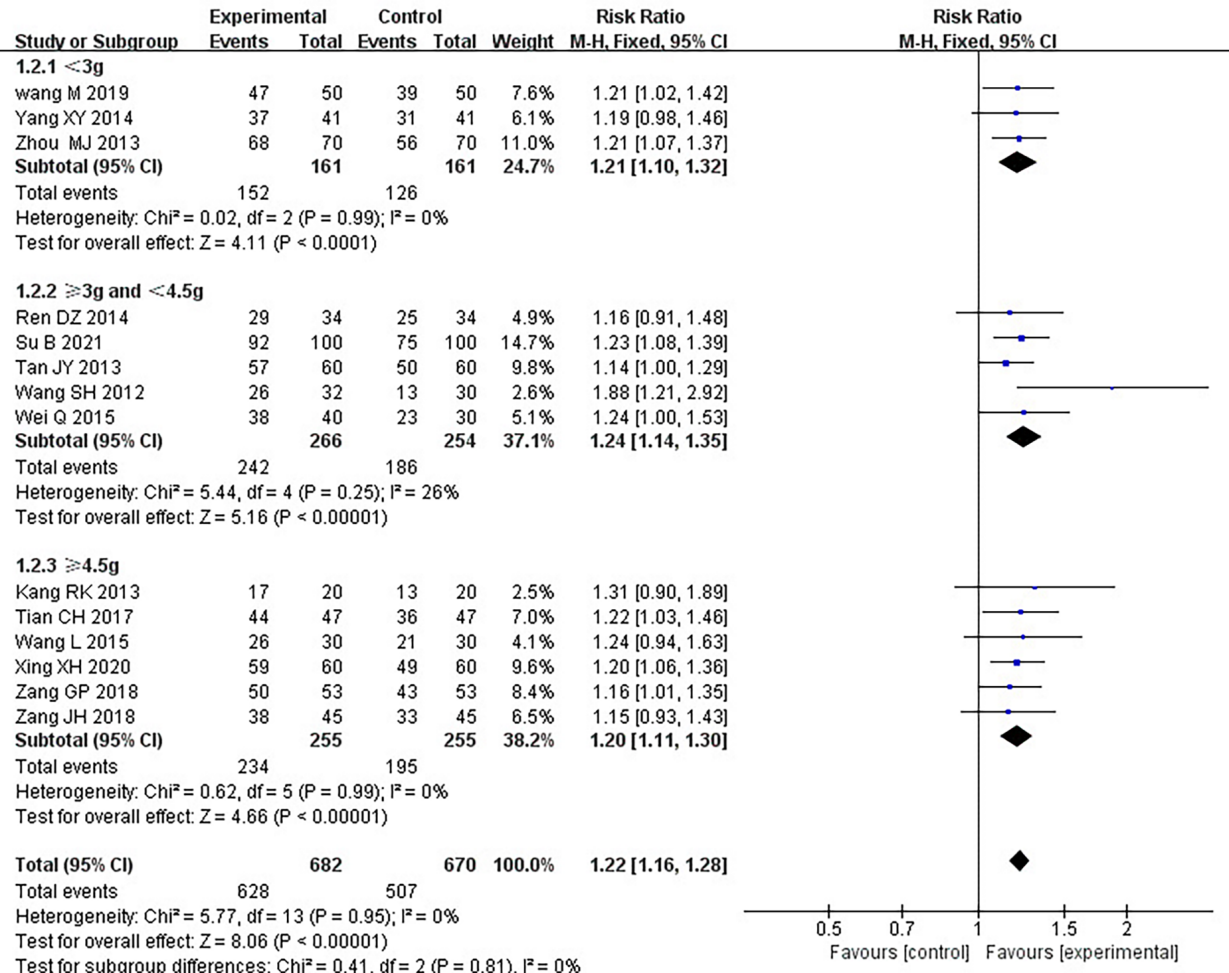
Sensitivity analysis of the duration of angina pectoris.

**Figure 5:**
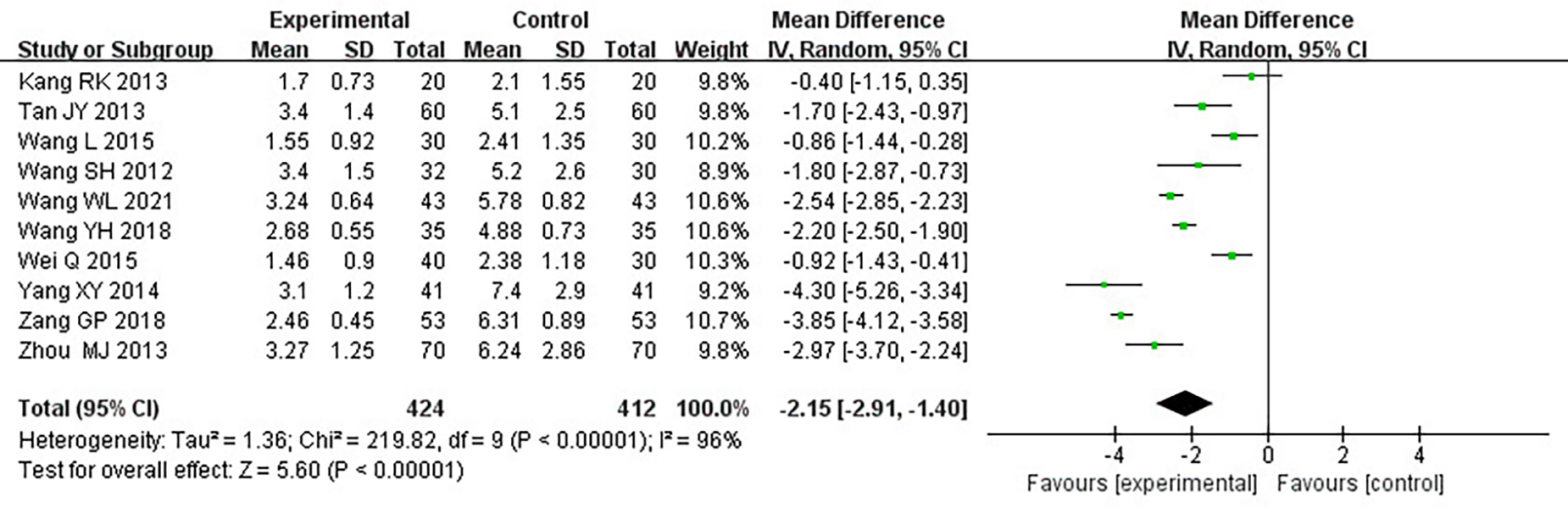
Sensitivity analysis of the degree of angina pectoris.

**Figure 6:**
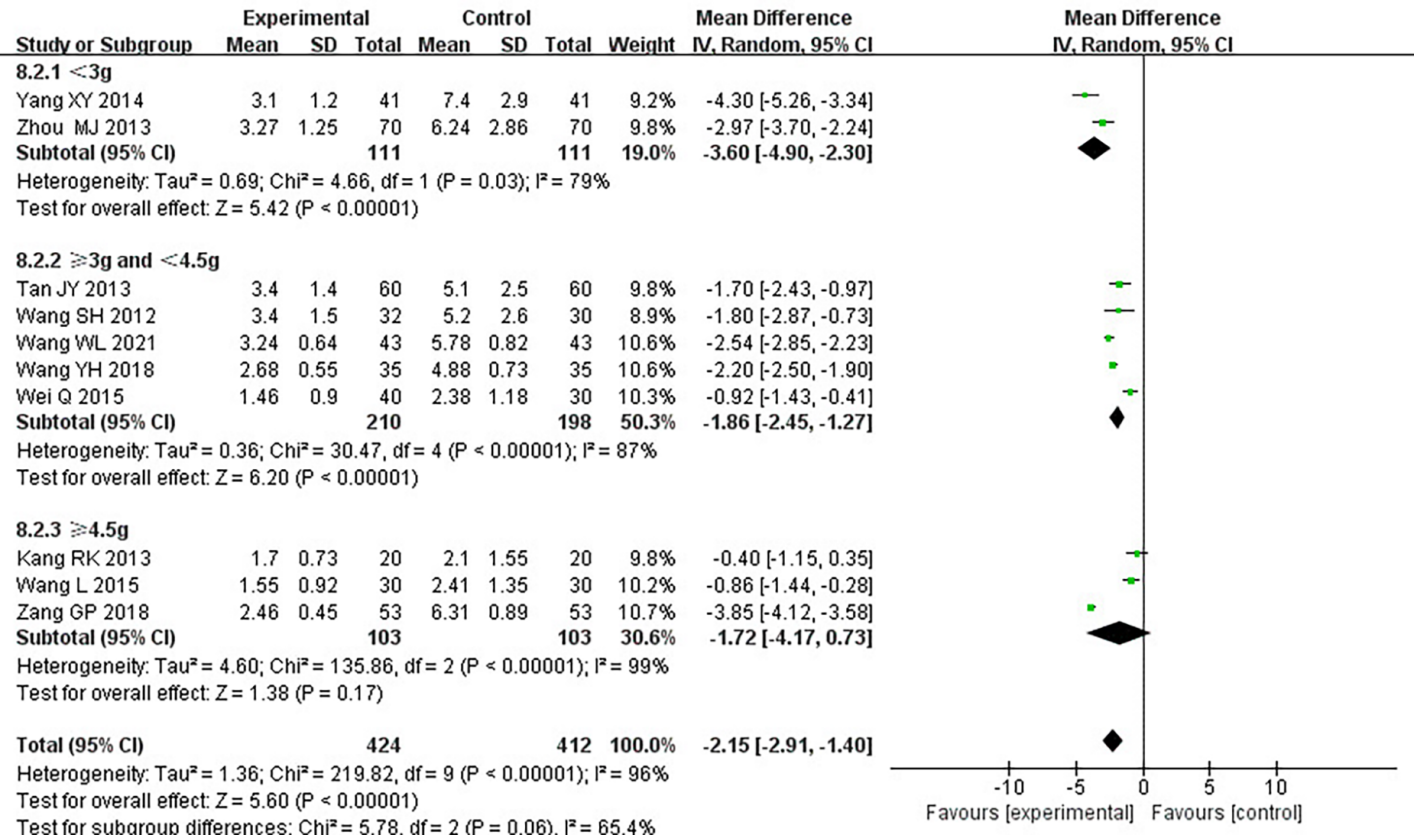
Sensitivity analysis of TC.

**Figure 7:**
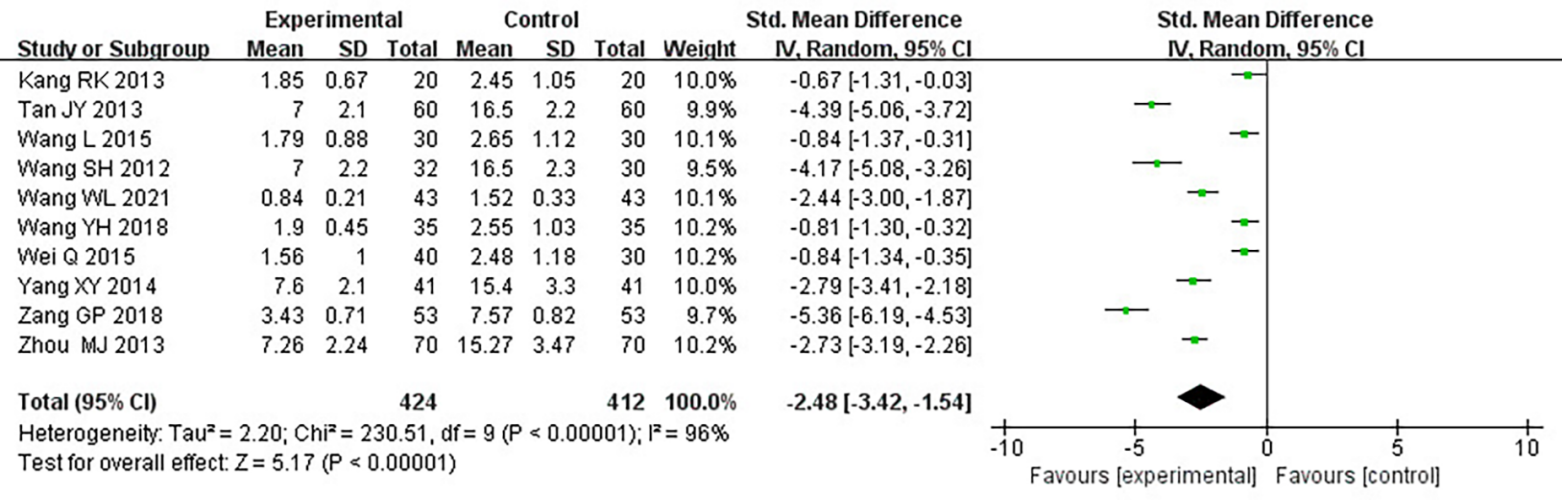
Sensitivity analysis of TG.

**Figure 8:**
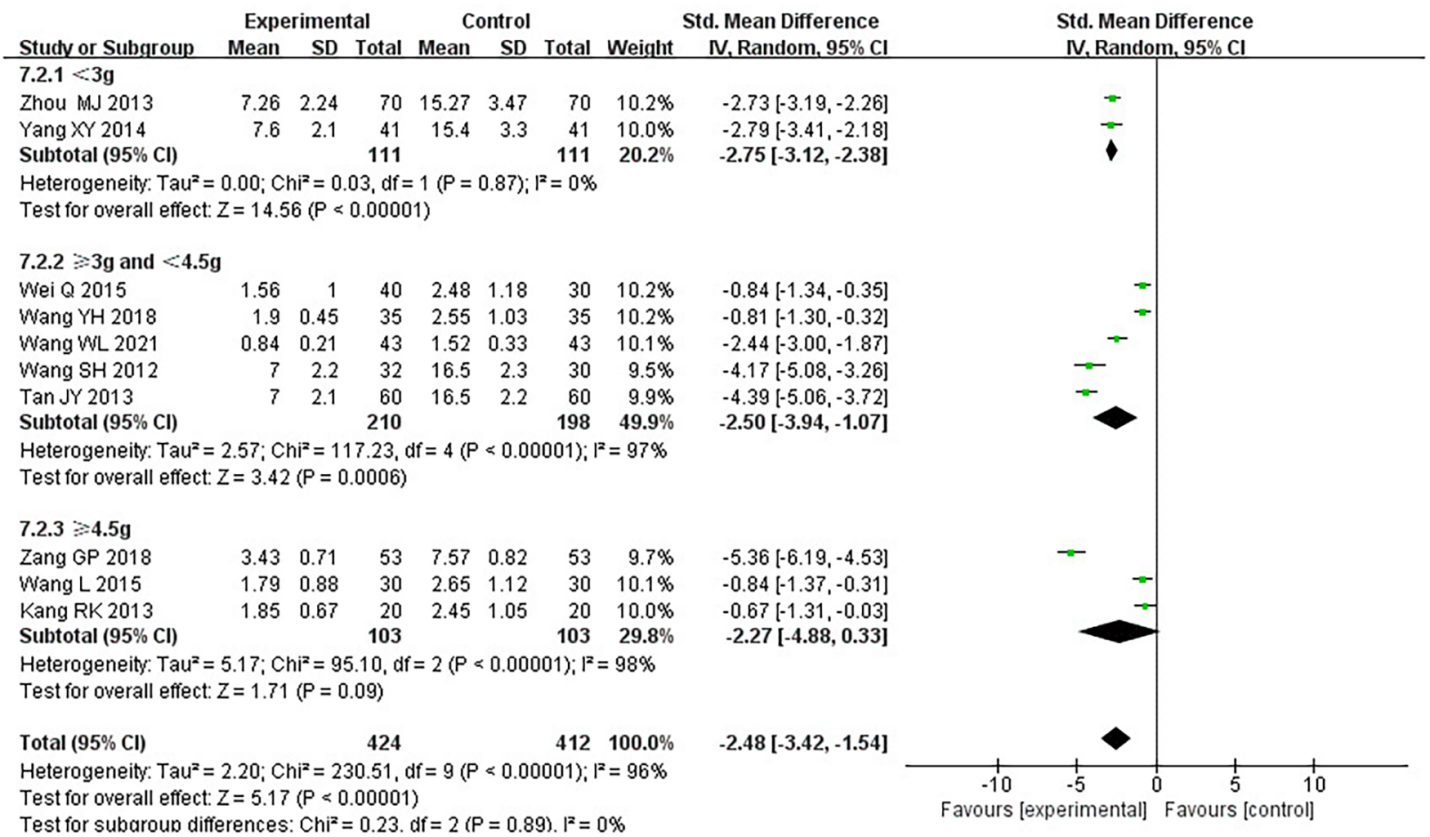
Sensitivity analysis of LDL-C.

**Figure 9:**
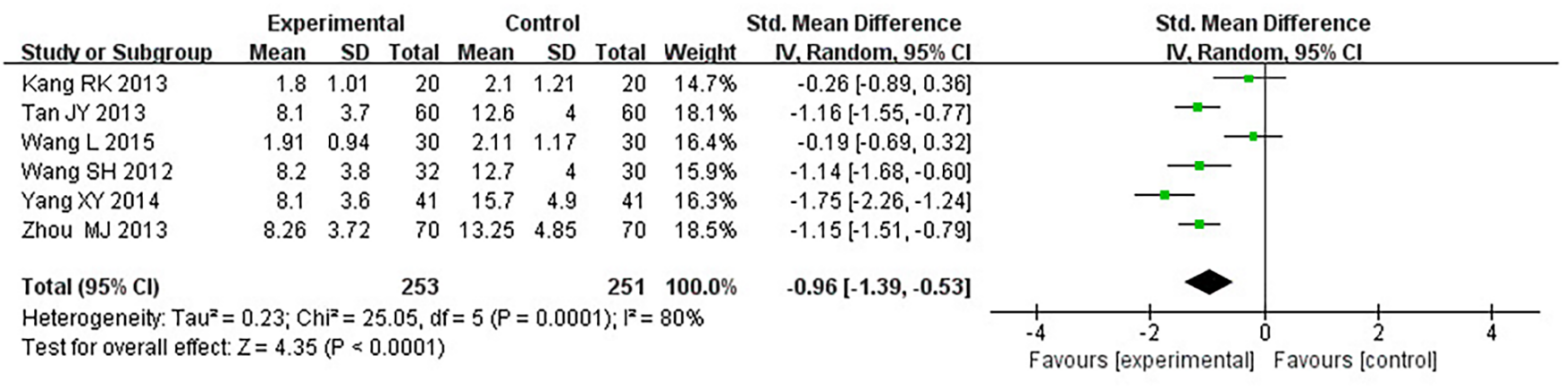
Sensitivity analysis of HDL-C.

**Figure 10:**
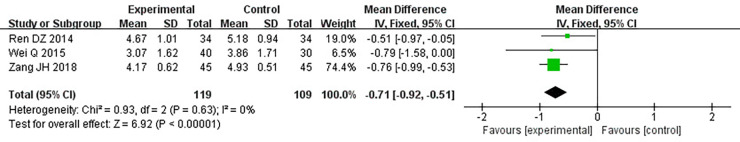
Sensitivity analysis of adverse events.

Through STATA.14 software, sensitivity analysis was conducted for all outcome indicators, including clinical treatment effect, improvement of angina pectoris (pain frequency, duration, and degree), blood lipid status (TC, TG, LDL-C, and HDL-C), and adverse events. Results showed no significant change in the size of the effect of the outcome indicators after excluding any study, indicating reliable and stable meta-analysis results (Figures 2–10).

### 3.6. Publication bias

**Figure 11:**
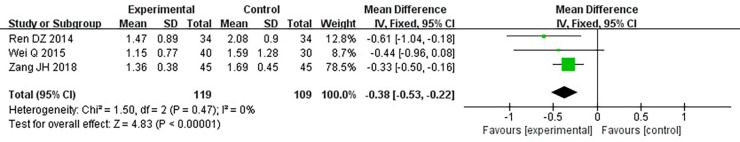
Funnel plot of clinical treatment effects.

A funnel plot detected publication bias in the primary outcome indicators. As shown in the figure, the asymmetric funnel plot indicated that publication bias might exist (Figure 11). Subsequently, Egger's and Begg's tests were used. Egger's test (*P *= 0.0726 > 0.05) suggested no publication bias, and Begg's test (*P *= 0.0487 < 0.05) suggested publication bias. To sum up, publication bias exists.

### 3.7. Overall quality of evidence by GRADE

The available evidence was evaluated using the GRADE method. Clinical treatment effect, improvement of angina pectoris (duration, frequency, and degree), and incidence of adverse events were rated as “low.” The downgraded contents included the following: (1) some studies did not describe randomization, and only one study described participants, personnel, and outcome assessments; and (2) publication bias from a funnel plot test was shown. Improvement of blood lipids (TC, TG, LDL-C, and HDL-C) was rated as “very low,” and the downgraded contents included the following: (1) some studies did not describe randomization, and only one study described participants, personnel, and outcome assessments; (2) due to the small number of studies included and the wide confidence interval, the downgrade was carried out; and (3) publication bias from a funnel plot test was shown (Table 18).

**Table 18:** GRADE summary table of outcome indicator evidence quality.

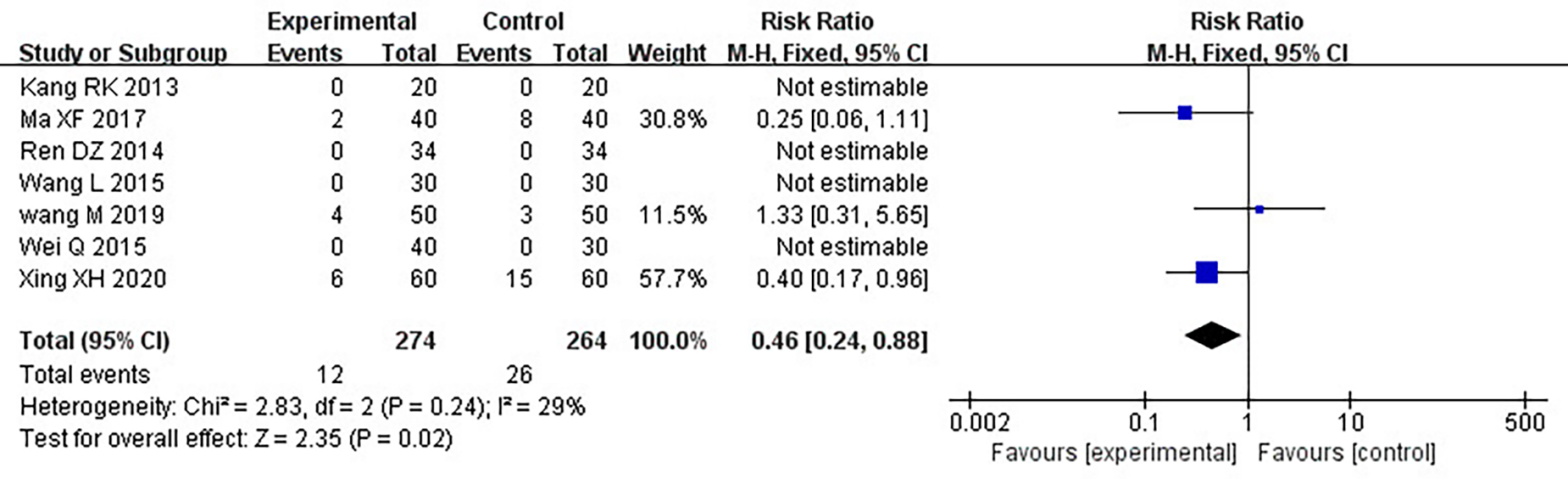

## 4. Discussion

The deposition of coronary artery lipids, the formation of atherosclerotic plaque, and disorders of lipid metabolism are involved in the pathogenesis of CHD. Therefore, while improving the myocardial blood supply, it is essential to regulate the concentration of blood lipids, enhance the cardiomyocyte's tolerance to ischemia, and improve the state of blood hypercoagulation (34). Guideline-recommended drugs such as aspirin, statins, angiotension converting enzyme inhibitors (ACEI)/angiotonin receptor blockers (ARBs), and *β*-blockers are widely used to prevent and treat CHD (35). However, the actual use situation and clinical efficacy are not optimistic (36, 37), related to individual patient differences, compliance, and adverse drug reactions (7, 38). Therefore, guideline-based standardized therapy should also consider individual patient differences, and seeking complementary or alternative therapies for CHD is necessary.

In the concept of TCM, CHD belongs to “Xiong bi” (chest obstruction) and “zhen xin tong” (absolute heart pain) and is the disease of intermingled deficiency and excess, with asthenia in origin and superficiality. The deficiency is dominated by qi deficiency, and the excess is dominated by blood stasis and phlegm turbidity (39, 40). Danlou tablets are composed of Danshen (*Radix Salviae Miltiorrhiae*), Gualou (*Fructrs Trichosanthis*), Gegen (*Radix Puerariae*), Chishao (*Radix Paeoniae Rubra*), Xiebai (*Bulbus Allii Macrostemi*), Chuanxiong (*Rhizoma Chuanxiong*), Yujin (*Radix Curcumae*), Zexie (*Rhizoma Alismatis*), Huangqi (*Radix Astragali*), and Gusuibu (*Rhizoma Drynariae*), which has the effect of relieving chest, dispelling phlegm, dispersing knot, and activating blood to remove stasis. Therefore, it plays a good role in treating CHD of blood stasis and phlegm turbidimetry syndromes (41). Experimental studies have fully exemplified that Danlou tablets can reduce myocardial ischemia and reperfusion injury (42), regulate procholesterol efflux, and perform anti-inflammation by activating the PPAR*α*/ABCA1 signaling pathway; concurrently, the NF-κB signaling pathway is prevented, thereby playing its role in alleviating atherosclerosis (13). The main chemical components of Danlou tablets include flavonoids, tanshinones, protostane triterpenoids, and paeoniflorin (43), which have antioxidant, antiplatelet aggregation, and antithrombosis effects and are the main bioactive compounds used to treat cardiovascular diseases (6).

Our results indicated a better effect of Danlou tablets combined with Western medicine than Western medicine alone in treating CHD, mainly in improving the clinical treatment effect, reducing the angina pectoris attack frequency, shortening the angina pectoris attack duration, reducing the angina pectoris degree, reducing TC, TG, and LDL-C levels, and improving HDL-C levels. We evaluated the quality of evidence for outcome indicators using the GRADE method. The quality of evidence for clinical treatment effect, improvement of angina pectoris (duration, frequency, and degree), and incidence of adverse events were “Low.” The quality of evidence regarding the improvement of blood lipids (TC, TG, LDL-C, and HDL-C) was “very low.” Clinical treatment efficacy is a common index for evaluating the TCM curative effect, which can reflect the overall therapeutic effect. The results showed a better clinical treatment effect of Danlou tablets combined with Western medicine than in treating CHD alone. Subgroup analysis results showed that a high dose (≥4.5 g per day) or a low dose (<4.5 g per day) of Danlou tablets could improve the therapeutic effect. Combined with the effect size, the efficacy of a low dose was better than that of a high dose. It can be seen from the results of the meta-analysis that adding Danlou tablets to conventional Western medicine treatment could improve the frequency, duration, and pain degree of angina pectoris. Subgroup analysis results displayed no difference in the angina pectoris frequency and the duration between the two groups when the dose of Danlou tablets was ≥4.5 g per day (*P* > 0.05). Since the observation time of one study (19) was 6 months, much longer than that of the other studies, we suspected that the treatment course affected the difference between the two groups. However, when we excluded this study, the results did not change, and the sensitivity analysis showed that excluding any of the studies would not change the robustness of the results. We conducted a subgroup analysis based on the course of treatment to explore the effects of different treatments on the results. The results showed the worst outcome for 180 days of treatment, and even no difference in efficacy from the control group, which may be associated with two studies of 180 days in the high-dose group (≥4.5 g per day) (19, 32). Therefore, low-dose Danlou tablets may have a better effect on angina pectoris. Dyslipidemia is a significant critical risk factor for CHD, and prevention and reasonable control of dyslipidemia can significantly change the morbidity and mortality of cardiovascular diseases (44, 45). Meta-analysis results showed that Danlou tablets had positive efficacy in reducing TC, TG, and LDL-C levels and improving HDL-C levels. Despite the high homogeneity of the study results and robust results by sensitivity analysis, only three studies reported changes in blood lipids with a small sample size; therefore, more large clinical studies are required to confirm this conclusion. Adverse events are crucial indicators to evaluate the feasibility of treatment. Meta-analysis results showed no increase in the incidence of adverse events from Danlou tablets, but it was not clear whether Danlou tablets could reduce the adverse reactions caused by Western drugs because most of the studies reporting adverse reactions were conducted for a short period (6 months in one study, 8 weeks in one study, and 4 weeks in the others). Adverse events were observed in only three studies, so more long-term follow-up studies are needed to evaluate the impact of Danlou tablets on adverse events.

Heterogeneity analysis indicated that the results of the heterogeneity test in regard to the frequency, duration, and degree of angina pectoris showed significant heterogeneity; although subgroup analysis based on the dose of the Danshen tablet was performed, the heterogeneity was not eliminated. We also analyzed heterogeneity through the course of treatment. Since one of the included studies (19) had a course of 6 months and the remaining studies had a course of 28 or 30 days, heterogeneity remained the same when we excluded the study with a long course. Therefore, the dose and course of treatment of Danlou tablets may not be the primary sources of heterogeneity. Through a detailed comparison of the characteristics of the included studies, it was found that differences in Western medicine treatment options may have brought about more pronounced heterogeneity since the types and doses of Western oral medicine were not wholly the same among patients in all studies, and some studies did not report the name and dose of western medicine. In addition, there was also a specific difference in the patients' ages, which ranged from 48.88 ± 5.01 to 69.93 ± 2.04, with a large span. Despite the heterogeneity of some outcome indicators, sensitivity analysis showed that all meta-analysis results were robust.

Our meta-analysis had the following limitations: (1) The Western medicine treatment regimens in all the studies were not identical, and the age span of the patients in the study was large, which may increase clinical heterogeneity. (2) The blind method and concealment of distribution concealment were not reported in most studies, which may lead to a bias in the efficacy of Danlou tablets. (3) A small sample size was included in most studies conducted in just one clinical trial center.

## 5. Conclusion

The current evidence suggests that the combination of Danlou tablets and Western medicine can enhance the efficacy of CHD and does not increase adverse events. However, because of the limited number and quality of the included studies, the results of our study should be treated with caution. Further large-scale RCTs are necessary to verify the benefits of this approach.
