# Supplementary material for: Efficacy and safety of Danlou tablets in traditional Chinese medicine for coronary heart disease: a systematic review and meta-analysis
Source: Front Cardiovasc Med. 2023 Jun 7;10:1100006. doi: 10.3389/fcvm.2023.1100006 (PMC10282777; doi:10.3389/fcvm.2023.1100006)
Supplement: Supplementary file 1 [file Table2.docx]

| Number | Search terms |
| --- | --- |
| #1 | Coronary heart disease [MeSH] |
| #2 | Coronary Diseases [Title/Abstract] |
| #3 | Coronary Heart Diseases [Title/Abstract] |
| #4 | Disease, Coronary Heart [Title/Abstract] |
| #5 | Heart Disease, Coronary [Title/Abstract] |
| #6 | Angina Pectoris [Title/Abstract] |
| #7 | #1 OR #2 OR #3 OR #4 OR #5 OR #6 |
| #8 | Danlou Tablet [Title/Abstract] |
| #9 | Dan-lou Tablet [Title/Abstract] |
| #10 | Dan Lou [Title/Abstract] |
| #11 | #8 OR #9 OR #10 |
| #12 | #7 AND #11 |
